# Demographics, Clinical Profile, and Outcome Correlations in Patients With Massive Cerebral Infarction: A Retrospective Study

**DOI:** 10.7759/cureus.91974

**Published:** 2025-09-10

**Authors:** Muthu Subramaniyan Shankar, Arivoli R, KA Ashraf Hussain, Kavi Sindhuja, Kouchikaa Palanisamy, Harshhni V L, Sashanga Arumugam, Vejey Shankar, Samyutha Balaa Venkatasubbaraman, Shahul Irfan

**Affiliations:** 1 Internal Medicine, Vinayaka Mission’s Kirupananda Variyar Medical College and Hospitals, Vinayaka Mission’s Research Foundation (Deemed to be University), Salem, IND; 2 General Medicine, Government Medical College Nagapattinam, Nagapattinam, IND; 3 General Medicine, Government Pudukkottai Medical College and Hospital, Pudukkottai, IND; 4 Internal Medicine, Melmaruvathur Adhiparasakthi Institute of Medical Sciences and Research, Melmaruvathur, IND; 5 General Practice, Nandha Medical College and Hospital, Erode, IND; 6 Obstetrics and Gynaecology, SRM Prime Hospital, Chennai, IND; 7 General Medicine, Government Medical College and Hospital Cuddalore, Chidambaram, IND; 8 Internal Medicine, Rajah Muthiah Medical College and Hospital, Chidambaram, IND; 9 Emergency Medicine, Leonard Hospital, Batlagundu, IND; 10 Internal Medicine, Periyar Government Hospital, Chennai, IND

**Keywords:** brain thrombectomy, india, massive infarct, middle cerebral artery infarction, stroke

## Abstract

Background: Massive cerebral infarction (MCI), typically affecting over two-thirds of a major cerebral artery territory, most often the middle cerebral artery (MCA), is among the most devastating forms of ischemic stroke. It leads to extensive neuronal death, malignant cerebral edema, and elevated intracranial pressure, frequently resulting in rapid clinical deterioration, herniation, and death. Understanding how demographic, clinical, and radiological factors influence prognosis in MCI is essential for improving care strategies and public health interventions.

Study: A retrospective observational study was conducted at Madras Medical College, Chennai, from May 2024 to May 2025, including 110 adult patients with radiologically confirmed MCI. Inclusion criteria were infarctions involving at least two-thirds of MCA, anterior cerebral artery (ACA), posterior cerebral artery (PCA), or combined territories, confirmed by CT or MRI. Data were collected on demographics, comorbidities, neurological status at presentation, radiological features, treatment modalities, complications, and discharge outcomes. Functional outcome was assessed using the modified Rankin Scale (mRS), with scores of 0-4 defined as a good outcome. Statistical analysis was performed using IBM SPSS Statistics, with chi-square tests and correlation analyses to determine associations between variables and outcomes. The primary objective was to identify clinical and radiological predictors of mortality and poor functional outcome.

Results: The mean age was 61.8 years; 58.2% were male, and 70.9% had hypertension. Only 23.6% of patients arrived at the hospital within six hours of symptom onset. Early presentation correlated significantly with favorable outcomes (p = 0.0008). MCA territory infarction was most common (70.9%), and midline shift >5 mm was present in 36.4%. Dominant hemisphere involvement and hemorrhagic transformation were also frequent and associated with worse outcomes. Neurologically, 38.2% had a GCS ≤8, and 45.5% had NIHSS ≥16. Lower GCS strongly predicted higher mortality. Conservative management was used in 67.3%, and only a minority underwent thrombolysis (7.3%) or thrombectomy (3.6%) due to late presentation. Decompressive hemicraniectomy was performed in 18.2% of patients. In-hospital complications were common-aspiration pneumonia (20%), urinary tract infections (16.4%), and sepsis (12.7%). At discharge, only 55% had good mRS scores (0-4), while 27.3% died during hospital stay. Poor outcome correlated significantly with advanced age, delayed arrival, midline shift, and low GCS.

Conclusion: MCI remains a neurological emergency with high mortality and disability. Early hospital arrival, younger age, and absence of midline shift significantly improved prognosis. Delayed presentation, severe baseline deficits, and radiological features such as hemorrhagic transformation and midline shift were key predictors of poor outcome. Limited use of reperfusion therapies due to late referrals underscores the urgent need for public education, improved emergency triage, and expanded access to neurocritical care. Strengthening early intervention strategies and minimizing in-hospital complications could substantially improve functional recovery in patients with MCI.

## Introduction

Massive cerebral infarction (MCI), most commonly involving the territory of the middle cerebral artery (MCA), represents one of the most devastating forms of ischemic stroke. It is characterised by extensive cerebral tissue necrosis, often affecting more than two-thirds of the MCA territory, leading to severe neurological deficits, malignant cerebral oedema, and high mortality [1,2]. The resultant mass effect and raised intracranial pressure can precipitate rapid neurological deterioration, herniation, and death, particularly in the absence of timely and aggressive intervention. Despite advances in acute stroke care, including reperfusion therapies and decompressive craniectomy, the prognosis in MCI remains poor, with survivors frequently experiencing significant long-term disability [1,2]. The burden of MCI is of particular concern in low- and middle-income countries, where delays in hospital presentation, limited access to neuroimaging, and restricted availability of neurosurgical services often contribute to suboptimal outcomes [3]. Demographic factors such as age, sex, and socioeconomic status, along with clinical variables including baseline neurological status, comorbidities, and infarct characteristics, have been shown to influence survival and functional recovery [1,2]. Early identification of high-risk patients through the analysis of these parameters is crucial for guiding clinical decision-making, prioritising intensive care resources, and counselling patients and their families about prognosis [1].

Several studies have explored the relationship between clinical presentation, radiological findings, and outcomes in ischemic stroke, but relatively fewer have focused specifically on MCI as a distinct clinical entity [2]. Furthermore, while some prognostic scoring systems exist, their applicability across diverse populations is variable, and population-specific data are essential for refining prognostic models [3]. In the Indian context, where stroke onset-to-door times are often prolonged and awareness regarding early symptoms remains low, understanding the demographic and clinical profiles of MCI patients could help in designing targeted public health interventions and improving acute management strategies [3]. Given this background, there is a need for retrospective studies that comprehensively evaluate the demographics, clinical characteristics, and outcomes of patients with MCI and examine correlations between these factors. Such data can not only contribute to local epidemiological understanding but also serve as a baseline for future prospective research aimed at improving survival and reducing disability [1-3]. This study seeks to address this gap by analysing the demographic distribution, clinical presentation, and outcome correlations in a cohort of patients with MCI admitted to a tertiary care centre over a defined period.

## Materials and methods

This retrospective observational study was conducted in the Department of Neurology and allied critical care units of Madras Medical College, Chennai, over a period of one year from May 2024 to May 2025. The primary aim was to evaluate the association between pre-hospital delays, radiological features such as midline shift and hemorrhagic transformation, and in-hospital outcomes in a South Indian cohort of patients with MCI. Secondary objectives include describing demographic and vascular risk factor profiles, examining the impact of in-hospital complications, and analysing utilisation of reperfusion therapies and decompressive hemicraniectomy in a resource-limited setting. Institutional Ethics Committee approval from Madras Medical College was obtained prior to initiation (no. EC/NEW/INST/2024/TN/0107), and the study adhered to the principles of the Declaration of Helsinki. All adult patients aged 18 years and above admitted with a diagnosis of MCI during the study period were included. MCI was defined radiologically as an acute ischemic stroke involving at least two-thirds of the territory of the MCA, anterior cerebral artery, posterior cerebral artery, or combined vascular territories, as confirmed on computed tomography or magnetic resonance imaging of the brain. While malignant cerebral infarction is traditionally defined in relation to MCA territory involvement, we adopted a broader radiological definition to also include anterior cerebral artery (ACA), posterior cerebral artery (PCA), and combined large-territory infarctions. This operational choice was made to reflect the spectrum of real-world presentations and to ensure that prognostic correlations could be studied across different large arterial territories. For MCA territory infarctions, associated midline shift greater than 5 mm, effacement of basal cisterns, or signs of malignant cerebral oedema were considered supportive imaging features. Midline shift was measured at the baseline admission CT scan (initial imaging window) at the level of the septum pellucidum, using the distance between the ideal and displaced midline. A displacement >5 mm was considered significant. All measurements were cross-verified by a senior neuroradiologist to minimise inter-observer variability. Patients with primary intracerebral haemorrhage, haemorrhagic infarction without extensive parenchymal ischemia, brainstem infarctions, or incomplete medical records were excluded from the analysis.

Data were retrieved from hospital medical records, radiology archives, and intensive care unit logs using a standardised data extraction form. Information collected included demographic details such as age, sex, place of residence, occupation, and socioeconomic status; vascular risk factors such as hypertension, diabetes mellitus, dyslipidaemia, atrial fibrillation, ischaemic heart disease, prior stroke or transient ischaemic attack, smoking, and alcohol use; and clinical presentation variables such as time from symptom onset to hospital arrival, Glasgow Coma Scale score [4] at admission, National Institutes of Health Stroke Scale score [5], presence of seizures, headache, vomiting, altered sensorium, and focal neurological deficits. Radiological findings recorded included the vascular territory involved, extent of infarction, presence of haemorrhagic transformation, degree of midline shift, involvement of the dominant hemisphere, and any associated vascular occlusion seen on CT or MR angiography. Treatment modalities were provided according to institutional protocols. Patients eligible for intravenous thrombolysis received alteplase at a dose of 0.9 mg/kg (maximum 90 mg) within the therapeutic window, provided there were no contraindications. Mechanical thrombectomy was performed in selected cases of large vessel occlusion based on vascular imaging and availability of resources. Decompressive hemicraniectomy was undertaken at the discretion of the neurosurgical team, usually within 48 hours of stroke onset in patients with malignant oedema and deteriorating neurological status. Osmotic agents such as mannitol and hypertonic saline were used for intracranial pressure management as per standard critical care protocols. Seizure prophylaxis and anti-oedema measures were also instituted where indicated. Details of management, whether conservative or surgical (including decompressive craniectomy), use of osmotic agents, anticoagulation or antiplatelet therapy, ventilatory support, and other intensive care measures were also documented. In-hospital complications were recorded systematically. Pneumonia was defined as the presence of new infiltrates on chest radiograph associated with fever, leukocytosis, or purulent sputum. Sepsis was defined according to Sepsis-3 criteria (suspected or proven infection with organ dysfunction). A urinary tract infection was diagnosed based on positive urine culture with clinical features, while deep vein thrombosis was confirmed by Doppler ultrasound. Pressure sores were identified by clinical examination. These definitions were applied consistently across all cases. Outcome measures assessed were in-hospital mortality, length of hospital stay, modified Rankin Scale score [6] at discharge, and complications such as aspiration pneumonia, urinary tract infection, sepsis, deep vein thrombosis, and pressure sores. For this study, a good outcome was defined as a modified Rankin Scale (mRS) score of 0-4 at discharge, representing survival with independence or partial dependence. Poor outcome was defined as mRS 5-6. The neurological scales (GCS, NIHSS, and mRS) used in the study are widely validated, have been described in their original publications, and are freely accessible for research purposes

Data entry was done using Microsoft Excel (Microsoft Corp., USA), and statistical analysis was performed with IBM SPSS Statistics for Windows, Version 25.0 (released 2017, IBM Corp., Armonk, NY). Continuous variables were expressed as mean with standard deviation for normally distributed data or median with interquartile range for skewed data, while categorical variables were presented as frequencies and percentages. Comparisons between categorical variables and outcomes were made using Chi-square or Fisher’s exact test, and continuous variables were compared using independent-sample t-tests or Mann-Whitney U tests as appropriate. Correlations between continuous predictors, such as midline shift with functional outcome, were evaluated using Pearson or Spearman correlation coefficients. A p-value less than 0.05 was considered statistically significant. The sample size was determined using the single-proportion formula n = Z^2 p(1-p)/d^2, with an anticipated in-hospital mortality of 40%, a 95% confidence level (Z = 1.96), and 10% absolute precision, which gave a required sample size of 93. As this was a retrospective study conducted from May 2024 to May 2025, a total of 110 eligible patients meeting the inclusion criteria were identified and included in the final analysis.

All eligible cases within the study period were included to minimise selection bias, and data abstraction was performed independently by two investigators with discrepancies resolved through discussion with a senior neurologist. Radiological parameters were cross-verified by a neuroradiologist to ensure accuracy. Given the retrospective nature of the study, individual patient consent was waived, and confidentiality was maintained by anonymising all identifiable patient information during data collection and analysis.

## Results

The study cohort comprised 110 patients with MCI, with a mean age of 61.8 ± 12.4 years. More than half of the patients were over 60 years of age, and males accounted for a slightly higher proportion than females (Table 1). Urban residents were more common than rural residents. Hypertension was the most prevalent vascular risk factor, followed by diabetes mellitus and dyslipidaemia. Atrial fibrillation, although less frequent, was present in one-fifth of patients. Smoking and alcohol use were observed in roughly one-third of the cohort, while ischaemic heart disease was the least common comorbidity.

**Table 1:** Demographic details of the patients. n = number of patients.

Parameter	Value (n, %)
50 years	18 (16.4%)
51–60 years	32 (29.1%)
>60 years	60 (54.5%)
Male	64 (58.2%)
Female	46 (41.8%)
Urban residence	68 (61.8%)
Rural residence	42 (38.2%)
Hypertension	78 (70.9%)
Diabetes mellitus	64 (58.2%)
Dyslipidaemia	48 (43.6%)
Atrial fibrillation	22 (20.0%)
Ischaemic heart disease	18 (16.4%)
Smoking	40 (36.4%)
Alcohol use	38 (34.5%)

Only 23.6% of patients presented to the hospital within six hours of symptom onset, while the majority arrived between six and 24 hours, and nearly one-third presented after 24 hours (Table 2). Early presentation was significantly associated with improved survival and functional outcomes, emphasising the importance of rapid recognition and referral in MCI.

**Table 2:** Distribution of patients based on their time of arrival to the hospital.

Onset-to-door time	Value (n, %)
≤6 hours	26 (23.6%)
>6–24 hours	48 (43.6%)
>24 hours	36 (32.7%)

The majority of patients (70.9%) had infarctions involving the MCA territory, followed by combined territory involvement in 20.0% of cases. ACA and PCA territory infarctions were relatively uncommon. Midline shift greater than 5 mm was observed in 36.4% of patients, while dominant hemisphere involvement was present in over half of the cohort (Table 3). Haemorrhagic transformation occurred in 16.4% of cases, often associated with larger infarct volumes and more severe neurological deficits.

**Table 3:** Distribution of patients based on the territiory of brain involved. MCA: middle cerebral artery, ACA: anterior cerebral artery, PCA: posterior cereberal artery

Radiological parameter	Value (n, %)
MCA territory infarction	78 (70.9%)
ACA territory infarction	6 (5.5%)
PCA territory infarction	4 (3.6%)
Combined territory infarction	22 (20.0%)
Midline shift > 5 mm	40 (36.4%)
Dominant hemisphere involvement	62 (56.4%)
Haemorrhagic transformation	18 (16.4%)

A substantial proportion of patients (38.2%) presented with a Glasgow Coma Scale (GCS) score of 8 or below, indicating severe neurological impairment (Table 4). Nearly half of the cohort had a National Institutes of Health Stroke Scale (NIHSS) score of 16 or higher, reflecting a high stroke severity burden. Altered sensorium was observed in 63.6% of patients, while seizures were present in 16.4%. Headache and vomiting were less common but still notable symptoms at admission.

**Table 4:** Distribution of the baseline neurological status of the patient. GCS: Glasgow Coma Scale, NIHSS: National Institute of Health Stroke Scale

Neurological parameter	Value (n, %)
GCS ≤ 8 at admission	42 (38.2%)
GCS > 8 at admission	68 (61.8%)
NIHSS score ≥ 16	50 (45.5%)
NIHSS score < 16	60 (54.5%)
Seizures at presentation	18 (16.4%)
Altered sensorium	70 (63.6%)
Headache	26 (23.6%)
Vomiting	34 (30.9%)

The majority of patients (67.3%) were managed conservatively, while 18.2% underwent decompressive craniectomy. Osmotic agents were administered to 60.0% of patients, and ventilatory support was required in 25.5% of cases (Table 5). Dual antiplatelet therapy was initiated in 43.6% of patients, while anticoagulation therapy was reserved for 10.9%, predominantly in cases with atrial fibrillation or other cardioembolic sources.

**Table 5:** Distribution of patients based on the predominant treatment received.

Management modality	Value (n, %)
Conservative management	74 (67.3%)
Decompressive craniectomy	20 (18.2%)
Osmotic agents used	66 (60.0%)
Ventilatory support	28 (25.5%)
Anticoagulation therapy	12 (10.9%)
Dual antiplatelet therapy	48 (43.6%)

The majority of patients (52.7%) had a hospital stay between six and 10 days, with equal proportions (23.6%) discharged within five days or staying beyond 10 days (Table 6). Prolonged hospitalisation was often associated with severe neurological deficits, medical complications, or the need for surgical intervention.

**Table 6:** Distribution of length of stay in the hospital.

Length of stay	Value (n, %)
≤5 days	26 (23.6%)
6–10 days	58 (52.7%)
>10 days	26 (23.6%)

Aspiration pneumonia was the most common complication, affecting 20.0% of the cohort, followed by urinary tract infections (16.4%) and sepsis (12.7%) (Table 7). Pressure sores and deep vein thrombosis were less frequent but still significant contributors to morbidity. The presence of complications was associated with poorer outcomes and longer hospital stays.

**Table 7:** Complication developed among the study population.

Complication	Value (n, %)
Aspiration pneumonia	22 (20.0%)
Urinary tract infection	18 (16.4%)
Sepsis	14 (12.7%)
Deep vein thrombosis	8 (7.3%)
Pressure sores	10 (9.1%)

Patients aged ≤60 years demonstrated a significantly higher rate of good outcomes compared to those older than 60 years (57.6% vs. 21.1%, p = 0.001), indicating that advanced age was strongly associated with increased mortality and disability. Sex distribution did not show a statistically significant correlation with outcome (p = 0.532). Residence (urban vs. rural) and the presence of hypertension or diabetes mellitus also did not reach statistical significance in predicting outcome.

A striking finding was the impact of hospital arrival time on prognosis. Patients presenting within six hours of symptom onset had markedly better outcomes (42.4% favourable) compared to those arriving later (10.5% favourable), with this association being highly significant (p = 0.0008) (Table 8). This highlights the importance of early recognition and prompt referral in improving survival and functional recovery in MCI.

**Table 8:** Comparison of outcomes based on their clinical profile. Fischer's exact test is used to calculate the p-value. p values <0.05 is considered as statistically significant.

Parameter	Good outcome n (%)	Poor outcome n (%)	p-value
Age ≤ 60 years	38 (57.6%)	12 (21.1%)	0.001*
Age > 60 years	28 (42.4%)	45 (78.9%)	
Male	36 (54.5%)	28 (49.1%)	0.532
Female	30 (45.5%)	29 (50.9%)	
Urban residence	40 (60.6%)	28 (49.1%)	0.187
Rural residence	26 (39.4%)	29 (50.9%)	
Hypertension	44 (66.7%)	34 (59.6%)	0.412
No hypertension	22 (33.3%)	23 (40.4%)	
Diabetes mellitus	38 (57.6%)	26 (45.6%)	0.198
No diabetes mellitus	28 (42.4%)	31 (54.4%)	
Onset-to-door ≤ 6 hours	28 (42.4%)	6 (10.5%)	0.0008*
Onset-to-door > 6 hours	38 (57.6%)	51 (89.5%)	
GCS ≤ 8	4 (18.2%)	38 (43.2%)	0.034*
GCS > 8	18 (81.8%)	50 (56.8%)	

Radiological analysis revealed that patients with MCA territory infarctions had significantly poorer outcomes compared to those with ACA or PCA territory involvement (p = 0.002 and p = 0.041, respectively). Combined territory infarctions were also associated with poorer outcomes (p = 0.015). Midline shift greater than 5 mm was a strong predictor of poor prognosis (p = 0.001), as was haemorrhagic transformation (p = 0.004) (Table 9). Dominant hemisphere involvement showed a statistically significant correlation with worse functional recovery (p = 0.047). 

**Table 9:** Comparison of outcomes based on radiological parameters. Fischer's exact test is used to calculate the p-value. p-values <0.05 are considered statistically significant. MCA: middle cerebral artery, ACA: anterior cerebral artery, PCA: posterior cereberal artery

Radiological parameter	Good outcome n (%)	Poor outcome n (%)	p-value
MCA territory infarction	12 (15.4%)	66 (84.6%)	0.002*
ACA territory infarction	4 (66.7%)	2 (33.3%)	0.041*
PCA territory infarction	3 (75.0%)	1 (25.0%)	0.083
Combined territory infarction	5 (22.7%)	17 (77.3%)	0.015*
Midline shift > 5 mm	6 (15.0%)	34 (85.0%)	0.001*
Dominant hemisphere involvement	18 (29.0%)	44 (71.0%)	0.047*
Haemorrhagic transformation	2 (11.1%)	16 (88.9%)	0.004*

Patients who underwent reperfusion therapies such as intravenous thrombolysis and mechanical thrombectomy had the highest proportion of good outcomes at discharge (62.5% and 75.0%, respectively), reflecting the benefits of timely vessel recanalisation in selected cases. Decompressive hemicraniectomy, while lifesaving in cases of malignant cerebral oedema, was associated with a lower rate of good outcomes (30.0%), largely due to the severity of the underlying infarction and preoperative neurological status. Patients managed conservatively with anti-oedema measures had good outcomes in only 25.6% of cases, highlighting the limitations of non-surgical interventions in MCI. Seizure medications were administered to patients with poorer baseline neurological function, reflected in the lower proportion of favourable outcomes (22.2%) (Table 10).

**Table 10:** Outcome in patients after receiving appropriate treatment.

Treatment modality	Good outcome (mRS 0–4)	Poor outcome (mRS 5–6)
Intravenous thrombolysis	5 (62.5%)	3 (37.5%)
Mechanical thrombectomy	3 (75.0%)	1 (25.0%)
Decompressive hemicraniectomy	6 (30.0%)	14 (70.0%)
Conservative management with anti-oedema measures	20 (25.6%)	58 (74.4%)
Seizure medications	4 (22.2%)	14 (77.8%)

## Discussion

MCI remains one of the most severe subtypes of ischemic stroke, with high mortality and long-term disability despite advances in acute stroke care. In our cohort, the in-hospital mortality rate was 27.3%, aligning with earlier studies that reported mortality ranging from 25% to 78% depending on patient selection and timing of intervention [7,8]. Age emerged as a significant prognostic factor, with patients over 60 years demonstrating markedly poorer outcomes, a finding supported by population-based studies that have consistently shown advanced age to be an independent predictor of mortality and severe disability following large territory infarcts [9,10]. The predominance of MCA territory involvement (70.9%) in our series is consistent with the vascular anatomy and epidemiology of large vessel occlusions, as MCA strokes are the most common and often result in extensive hemispheric injury [11]. Our data also confirmed that dominant hemisphere involvement was associated with worse outcomes, possibly due to the additional burden of language and higher cognitive deficits, as has been reported in prior observational studies [12]. It is important to note that most prior studies have used the term malignant MCA infarction, whereas our study applied a broader definition encompassing ACA, PCA, and combined large-territory infarctions. This methodological decision, while limiting direct comparability with malignant MCA series, enhances external validity by reflecting the heterogeneity of massive infarctions encountered in clinical practice. Including such cases may improve the generalisability of prognostic determinants across diverse populations.

Delayed hospital presentation was frequent, with only 23.6% arriving within six hours of onset. This delay significantly reduced the likelihood of a favourable outcome, which concurs with evidence that early hospital arrival improves eligibility for reperfusion therapies and allows timely initiation of neuroprotective strategies [13,14]. Radiologically, midline shift greater than 5 mm was strongly correlated with unfavourable outcomes, in agreement with previous analyses demonstrating that the extent of mass effect is a robust predictor of mortality due to its association with malignant cerebral oedema and herniation risk [15]. Haemorrhagic transformation was observed in 16.4% of our cases, which is within the range reported in large infarct cohorts, and is recognised as a marker of severe reperfusion injury and poor neurological prognosis [16]. The prevalence of vascular risk factors such as hypertension (70.9%) and diabetes (58.2%) in our cohort underscores the ongoing challenge of primary prevention, as these comorbidities are well-established contributors to both the occurrence and the unfavourable course of ischemic stroke [17,18].

Correlation analysis in our study revealed that baseline neurological severity, as measured by the GCS, was strongly associated with in-hospital outcomes. Patients with GCS ≤8 had a markedly higher mortality and disability rate compared to those with higher scores, reflecting the established prognostic value of early consciousness level in large hemispheric infarcts [9,15]. Similar trends have been reported in pooled analyses, where low admission GCS independently predicted fatal cerebral oedema and poor functional recovery, irrespective of age and infarct volume [7,15]. Radiologically, ACA territory involvement, although less common than MCA infarction, was linked to worse outcomes when combined with large MCA or PCA infarctions, supporting the hypothesis that multi-territorial involvement reflects more extensive arterial occlusion and impaired collateral circulation [11,19]. Midline shift magnitude correlated significantly with mortality, underscoring the impact of severe cerebral mass effect, which aligns with previous literature [15,20].

Complication rates in our cohort were notable, with aspiration pneumonia (27.3%), urinary tract infections (21.8%), and sepsis (12.7%) contributing to extended hospital stay and worsened functional outcomes. These findings parallel earlier multicentre studies demonstrating that medical complications during stroke admission significantly increase dependency at discharge and one-year mortality [18,21]. The observed rate of haemorrhagic transformation (16.4%) was comparable to prior prospective reports [18], and such events were disproportionately seen in patients with delayed presentation, larger infarct volumes, and those who underwent surgical decompression. While decompressive hemicraniectomy is known to improve survival in selected patients under 60 years [8], our analysis suggests that survival benefit may be offset by a higher proportion of severely disabled survivors in older age groups, aligning with findings from the DESTINY II trial [22]. This highlights the ethical and rehabilitative considerations in surgical decision-making for elderly patients with malignant infarction.

When compared with published regional and international data, the demographic profile in our cohort - predominance of patients over 60 years, male excess, and high prevalence of hypertension and diabetes - aligns with stroke epidemiology trends in South and East Asia [3,9,12]. The mean age in our series was slightly lower than that reported in large Western cohorts [8,13], a pattern also observed in other Indian series, possibly reflecting earlier onset of vascular risk factor clustering and lower baseline awareness [3,12]. The high proportion of urban patients may be a function of our hospital’s tertiary referral status and urban location, but it also reflects increasing stroke incidence in urbanising populations [3].

The mortality rate of 27.3% observed in our cohort is lower than historical reports of malignant MCA infarction mortality exceeding 50% without surgical intervention [7,10,20], but remains higher than rates seen in centres with widespread early decompressive surgery and comprehensive stroke units [8,22]. Our data reinforce previous findings that outcome disparities are largely driven by delays in presentation, limited access to neurosurgical care, and high complication rates [3,15,18,21]. The strong association between delayed onset-to-door times and poor prognosis mirrors prior work from both Indian [3] and international settings [17], underscoring the urgent need to strengthen pre-hospital triage, public education on stroke warning signs, and timely interfacility transfers.

Recent randomised controlled trials such as DAWN [23] and DEFUSE-3 have extended the therapeutic window for mechanical thrombectomy in carefully selected patients, reshaping the prognosis of large vessel occlusions. However, in our cohort, only a small proportion underwent reperfusion therapy, reflecting real-world limitations in eligibility and access in low-resource settings. Furthermore, while updated AHA/ASA consensus statements emphasise early triage, decompressive surgery, and standardised stroke systems of care, implementation remains variable in India, contributing to delays and outcome disparities

From a systems perspective, our findings have direct implications for stroke care pathways in India. Early neuroimaging capacity, multidisciplinary acute stroke teams, and pre-defined criteria for surgical referral, elements emphasised in global consensus statements, remain unevenly implemented in low- and middle-income country settings [20]. Integrating these measures, along with proactive complication prevention strategies [18,21], could potentially improve both survival and functional outcomes in MCI. While our retrospective design precludes establishing causality, the correlations between clinical, radiological, and outcome parameters presented here provide a strong foundation for prospective validation and for informing locally adapted clinical protocols.

Strengths of the study

This study provides a comprehensive evaluation of MCI by analysing demographic, clinical, radiological, and outcome correlations in a reasonably large cohort of 110 patients. The inclusion of all eligible cases during the study period minimised selection bias and improved representativeness. Another strength is the detailed documentation of both clinical and radiological parameters, which were cross-verified by neurologists and neuroradiologists, enhancing the accuracy and reliability of findings. The study also assessed a wide spectrum of outcomes, including functional status at discharge, in-hospital mortality, complications, and management strategies, allowing for a holistic understanding of prognostic determinants. Furthermore, statistical methods were appropriately applied, with correlation analyses identifying independent predictors of poor outcomes, thereby generating clinically relevant insights. Importantly, the study highlights region-specific challenges, such as delayed hospital arrival and limited access to advanced interventions, which adds valuable epidemiological data to the Indian context, where the literature on MCI remains limited.

Limitations of the study

The primary limitation of this study is its retrospective design, which restricts the ability to establish causal relationships and introduces potential biases due to incomplete or inconsistent medical records. Being a single-centre study, the findings may not be generalisable to other regions, particularly rural or resource-limited settings, and may also reflect referral bias associated with a tertiary care centre. Outcome assessment was confined to in-hospital mortality and discharge functional status, without long-term follow-up such as 90-day mRS, which is the standard measure for functional recovery and quality of life after stroke. Advanced imaging modalities, including perfusion studies, were not uniformly available, limiting detailed evaluation of collateral circulation and penumbral tissue. We used a broader operational definition of MCI that included ACA, PCA, and multi-territory infarctions in addition to malignant MCA strokes, which may reduce direct comparability with studies restricted to classical malignant MCA infarction. We did not calculate infarct volumes, and infarct extent was assessed only by territory involvement on imaging. Midline shift was assessed only on baseline admission imaging, and serial or peak measurements over the course of cerebral oedema were not evaluated, which may underestimate its full prognostic significance. In addition, while the sample size was sufficient for basic correlations, it was not adequately powered for subgroup analyses of less common infarction types (such as ACA or PCA involvement), increasing the risk of type II error. Our statistical analysis was restricted to bivariate comparisons, and the lack of multivariate regression limited the ability to adjust for confounders and identify independent predictors. Hence, these associations should be interpreted as correlations rather than causative predictors. Socioeconomic determinants, rehabilitation strategies, and variations in access to interventions were not extensively evaluated, which may have influenced outcomes but remained unaccounted for. Our study is limited by its relatively modest sample size and descriptive design, which may not yield novel prognostic criteria beyond those established in larger series; however, it provides region-specific data and highlights real-world outcomes in a resource-limited setting. These limitations, taken together, highlight the need for larger, multicentre, prospective studies incorporating advanced imaging, long-term follow-up, and multivariate modelling to generate more robust and generalisable evidence.

## Conclusions

MCI remains a neurological emergency associated with substantial mortality and long-term disability despite advances in acute stroke management. In our cohort, advanced age, delayed hospital presentation, severe baseline neurological impairment, extensive radiological involvement, particularly midline shift and haemorrhagic transformation, and the occurrence of in-hospital complications emerged as key determinants of poor outcome. Conversely, younger age, early presentation within the therapeutic window, and absence of major mass effect were associated with improved survival and functional recovery.

These findings highlight the critical importance of early recognition, rapid referral, and timely neuroimaging to facilitate prompt intervention, including consideration for decompressive craniectomy where indicated. Strengthening public awareness of stroke symptoms, improving pre-hospital care systems, and enhancing access to specialised neurocritical care are essential to reduce delays and optimise management. In addition, vigilant prevention and management of medical complications during hospitalisation can further improve prognosis. Future multicentre, prospective studies are warranted to validate these results and develop region-specific protocols aimed at improving both survival and quality of life for patients with MCI.
